# An Automated Deductive Verification Framework for Circuit-building Quantum Programs

**DOI:** 10.1007/978-3-030-72019-3_6

**Published:** 2021-03-23

**Authors:** Christophe Chareton, Sébastien Bardin, François Bobot, Valentin Perrelle, Benoît Valiron

**Affiliations:** Imperial College, London, UK; 9 LMF, CentraleSupélec, Université Paris-Saclay, Gif-sur-Yvette, France; 10 CEA, LIST, Université Paris-Saclay, Palaiseau, France

**Keywords:** deductive verification, quantum programming, quantum circuits

## Abstract

While recent progress in quantum hardware open the door for significant speedup in certain key areas, quantum algorithms are still hard to implement right, and the validation of such quantum programs is a challenge. In this paper we propose Qbricks, a formal verification environment for circuit-building quantum programs, featuring both parametric specifications *and* a high degree of proof automation. We propose a logical framework based on first-order logic, and develop the main tool we rely upon for achieving the automation of proofs of quantum specification: PPS, a parametric extension of the recently developed path sum semantics. To back-up our claims, we implement and verify parametric versions of several famous and non-trivial quantum algorithms, including the quantum parts of *Shor’s integer factoring*, quantum phase estimation (QPE) and Grover’s search.

### 1 Introduction

**1.1 Quantum computing.** Quantum programming is seen as a potential revolution for many computing applications: cryptography [61], deep learning [7], optimization [23,22], solving linear systems [33], etc. In all of these domains, current quantum algorithms beat the best known classical algorithms by either quadratic or even exponential factors. In parallel to the rise of quantum algorithms, the design of quantum hardware has moved from lab-benches [14] to programmable, 50-qubits machines designed by industrial actors [4,38] reaching the point where quantum computers beat classical computers for specific tasks [4]. This has stirred a shift from a theoretical standpoint on quantum algorithms to a more programming-oriented view with the question of their concrete coding and implementation [66,65,55].

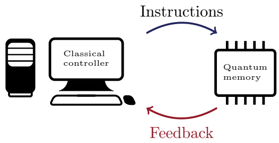

Fig. 1: The hybrid model

*In this context, an important problem is the adequacy between the mathematical description of an algorithm and its concrete implementation as a program.*

**1.2 The hybrid model.** The vast majority of quantum algorithms are described within the *quantum co-processor model* [42], i.e. a hybrid model where a *classical* computer controls a *quantum* co-processor holding a quantum memory (cf. Figure 1). The co-processor is able to apply a fixed set of elementary operations (buffered as *quantum circuits*) to update and query (*measure*) the quantum memory. Importantly, while measurement allows one to retrieve classical (probabilistic) information from the quantum memory, it also modifies it (*destructive effect*). The quantum memory state is represented by a linear combination of possible concrete values, generalizing the classical notion of probabilities to the complex case, and the core of a quantum algorithm consists in successfully setting the memory in a specific *quantum state.*

Major *quantum programming languages* such as Quipper [30], Liqui$|\rangle$ [67], Q# [64], ProjectQ [63], Silq [8], and the rich ecosystem of existing quantum programming frameworks [55] follow this hybrid model. Such *circuit-building quantum languages* are the current consensus for high-level executable quantum programming languages.

**1.3 The problem with quantum algorithms.** Starting from an initial state, a quantum algorithm typically describes a series of high-level operations which, once composed, realize the desired state. Each high-level operation may itself be described in a similar way, until one reaches elementary operations (*quantum gates*). Describing an algorithm therefore requires both to list these elementary operations, or *quantum circuit*, and to specify the circuit's behavior.

*A major issue is then to verify that the quantum circuit generated by the code written as an implementation of a given algorithm is indeed a run of this algorithm, and that the circuit has indeed the specified characteristics of shape (for instance: a polynomial size).*

While testing and debugging are the common verification practice in classical programming, they become extremely complicated in the quantum case: debugging and assertion checking are problematic due to the destructive aspect of quantum measurement, the probabilistic nature of quantum algorithms seriously impedes system-level quantum testing, and classical emulation of quantum algorithms is (strongly believed to be) intractable. On the other hand, nothing prevents *a priori* the formal verification [15] of quantum programs.

**1.4 Goal and challenges.** *Our goal is to provide an automated formal verification framework for circuit-building quantum programs.* Such a framework should satisfy the following principles: (1) *Parametricity:* it should allow parametric (i.e. scale-invariant) specifications and proofs, so as to enable the generic specification and verification of parametrized implementations. This is crucial as quantum algorithms always describe *parametrized families* of circuits; (2) *Proof automation:* it should, as far as possible, provide automatic proof means in order to ease adoption.

*Prior works on quantum formal verification do not fully reach these goals together, as they are either not parametric, or not automated.* Model-checking methods [27,70] are fully automatic but not parametric – moreover they are highly scale-sensitive. Recently, Amy [1,2] developed a powerful framework for reasoning over quantum circuits, the *path-sums symbolic representation*. Thanks to their good compositional properties, reasoning with path-sums is well automated and can scale up to large problem instances (up to 100 qubits). Yet, the method is not parametric and only addresses fixed-size circuits. On the other side of the spectrum, several approaches deal with parametricity but sacrifice automation as they generate proof obligations in higher-order logic, supported with proof assistants such as Coq or Isabelle/HOL. One can cite the approach of Boender et al. [10], Qwire [53,58], SQIR [35,34] or QHL [68,71,46,69,45]. Combined with the use of the standard *matrix semantics* for quantum circuits — that we show in Section 8 cumbersome for automation — only very few realistic quantum programs have been verified in a parametric way [45,35,34].

**1.5 Proposal.** We propose Qbricks, an automated formal verification framework for circuit-building quantum programs, featuring parametric specification *together* with a high degree of proof automation.

We bring two key innovations along the road: **(Key 1)** we propose the new *parametrized path-sums (PPS)* symbolic representation of families of quantum circuits, extending path-sums [1] to the parametric case while keeping good compositional properties. PPS prove extremely useful both as a specification mechanism and as an automation mechanism; (**Key 2**) we carefully tune together our programming language (Qbricks-DSL) and specification logic (Qbricks-Spec) so that the corresponding verification problem remains automatable in practice — first-order proof obligations — while the framework is still expressive enough to write, specify and verify realistic quantum programs (Shor order finding — Shor-OF [61], QPE [41,16], Grover [31]).

**1.6 Contributions.** We bring the following contributions.

- A flexible symbolic representation for reasoning about quantum states, building upon the recent path-sum symbolic representation [1,2]. Our representation, called *parametrized path-sums (PPS)*, retains the compositional and closure properties of regular path-sums while allowing *genericity* and *parametricity* of both specifications and proofs. Especially, first-order logic together with PPS provide a unified and powerful way to reason about many essential quantum concepts (Section 5.2) and fit well with the standard way of describing quantum algorithms. We are the first to highlight this connection and make PPS a "first-class" concept, where prior works are limited to standard path sums, or rely on the standard matrix semantics;
- A programming and verification framework, that is: on one hand, a core domain-specific language (Qbricks-DSL, Section 4) for describing families of quantum circuits, with enough expressive power to describe parametric circuits from non-trivial quantum algorithms; and on the other hand, a *first-order* logical (domain-specific) specification language (Qbricks-Spec, Section 5), tightly integrated with PPS and Qbricks-DSL to specify properties of parametrized programs representing families of quantum circuits. The careful interplay between these two components yields first-order proof obligations, and thus is a key aspect of proof automation;

- A dedicated proof engine: we introduce the Hybrid Quantum Hoare Logic (HQHL) deduction system for deductive verification over circuit-building quantum programs. It is tightly coupled with PPS and produces proof obligations in the Qbricks-Spec logic (Section 6);
- This framework is embedded in the Why3 deductive verification tool [9,24] as a DSL (Section 7), and provides proof automation mechanisms dedicated to the quantum case. This material is grounded in standard mathematics theories (linear algebra, arithmetic, complex numbers, binary operations, *etc.*) with 450+ definitions and 1,000+ lemmas. *All lemmas have been proved in Why3*, and the whole framework is publicly available;
- We present in Section 8 *the first ever verified parametric implementation of the quantum part of Shor's factoring algorithm* [61] (Order Finding, including the polynomial complexity of the circuits produced by our implementation and probability requirements), as well as verified parametric implementations of other major quantum algorithms: Quantum Phase Estimation (QPE) [41,16][3], Grover's (search) algorithm [31] and Quantum Fourier Transform (QFT) [18]. Our method achieves a high level of proof automation (96% on Shor-OF), significantly reducing proof effort (factor 13.6x vs. QHL on Grover, factors 7.7x and 6.4x vs. SQIR on resp. QPE and Grover).

Additional technical material can be found in the online extended versione [13]. Implementation and benchmarks are available online[54].

**1.7 Discussion.** The scope of this paper is limited to proving properties of circuit-building quantum programs. We do not claim to support right now the interactions between classical data and quantum data (referred to as "classical control" in the literature), nor the probabilistic side-effect resulting from the measurement. Still, we are already able to target realistic implementations of famous quantum algorithms, and thanks to equational theories for complex and real number we can automatically *reason* on the probabilistic outcome of a measurement. Also, we do not claim any novelty in the proofs for Shor-OF, QPE or Grover by themselves, but rather the first *highly-automated* parametric correctness proofs of the circuits produced by programs implementing them, and the first parametric correctness proofs of an implementation of Shor-OF.

### 2 Background: Quantum Algorithms and Programs

While in classical computing, the state of a bit is either 0 or 1, in quantum computing [50] the state of a *quantum bit* (or *qubit*) is described by *amplitudes* over the two elementary values 0 and 1 (denoted in the Dirac notation with $|0\rangle$ and $|1\rangle$), i.e. linear combinations $\alpha_0|0\rangle + \alpha_1|1\rangle$ where $\alpha_0$ and $\alpha_1$ are any *complex values* satisfying $|\alpha_0|^2 + |\alpha_1|^2 = 1$. In a sense, amplitudes are *generalization of probabilities*. More generally, the state of a *qubit register* of $n$ qubits

[3] QPE is a major quantum building block, at the heart of, e.g., HHL [33] logarithmic linear system solving algorithm or quantum simulation [28].

("qubit-vector") is any *superposition* of the $2^n$ elementary bit-vectors ("basis element", where a bit-vector $k \in \{0..2^n - 1\}$ is denoted $|k\rangle_n$), that is any $|u\rangle_n = \sum_{k=0}^{2^n-1} \alpha_k |k\rangle_n$ such that $\sum_{k=0}^{2^n-1} |\alpha_k|^2 = 1$. For example, in the case of two qubits, the basis is $|00\rangle$, $|01\rangle$, $|10\rangle$ and $|11\rangle$ (also abbreviated $|0\rangle_2$, $|1\rangle_2$, $|2\rangle_2$ and $|3\rangle_2$). Such a (*quantum state*) vector $|k\rangle_n$ is called a *ket* of length $n$ (and dimension $2^n$).

Technically speaking, we say that the quantum state of a register of $n$ qubits is represented by a normalized vector in a Hilbert space of finite dimension $2^n$ (a.k.a. finite-dimensional Hilbert space), whose basis is generated by the Kronecker product (a.k.a. tensor product, denoted $\otimes$) over the elementary bit-vectors. For instance, for $n = 2$: $|0\rangle \otimes |0\rangle$, $|0\rangle \otimes |1\rangle$, $|1\rangle \otimes |0\rangle$ and $|1\rangle \otimes |1\rangle$ act as definitions for $|00\rangle$, $|01\rangle$, $|10\rangle$ and $|11\rangle$.

**2.1 Quantum data manipulation.** The core of a quantum algorithm consists in manipulating a qubit register through two main classes of operations. (1) *Quantum gate.* Local operation on a fixed number of qubits, whose action consists in the application of a *unitary map* to the corresponding quantum state vector i.e. a linear and bijective operation preserving norm and orthogonality. The fact that unitary maps are bijective ensures that every unitary gate admits an *inverse.* Unitary maps over $n$ qubits are usually represented as $2^n \times 2^n$ *matrices.* (2) *Measurement.* The retrieval of classical information out of the quantum memory. This operation is probabilistic and modifies the state of a quantum register: measuring the $n$-qubit system $\sum_{k=0}^{2^n-1} \alpha_k |k\rangle_n$ returns the bit-vector $k$ of length $n$ with probability $|\alpha_k|^2$. Quantum gates might be applied in *sequence* or in *parallel*: sequence application corresponds to *map composition* (or, equivalently, matrix multiplication), while parallel application corresponds to the *Kronecker product*, or tensor product, of the original maps — or, equivalently, the Kronecker product of their matrix representations.[4]

**2.2 Quantum circuits.** In a way similar to classical Boolean functions, the application of quantum gates can be written in a diagrammatic notation: *quantum circuits.* Qubits are represented with horizontal wires and gates with boxes. Circuits are built *compositionally*, from a given set of *atomic gates* and by a small set of *circuit*

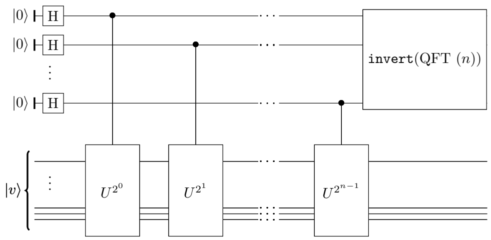

Fig. 2: The circuit for QPE

[4] Given two matrices $A$ (with $r$ rows and $c$ columns) and $B$, their Kronecker product is the matrix $A \otimes B = \begin{pmatrix} a_{11}B & \dots a_c B \\ \vdots & \ddots & \vdots \\ a_{r1}B & \dots a_{rc}B \end{pmatrix}$. This operation is central in quantum information representation. It enjoys a number of useful algebraic properties such as associativity, bilinearity or the equality $(A \otimes B) \cdot (C \otimes D) = (A \cdot C) \otimes (B \cdot D)$, where $\cdot$ denotes matrix multiplication.

*combinators*, including: parallel and sequential compositions, circuit inverting, controlling, iteration, ancilla creation, etc. As an example of a quantum circuit, we show in Figure 2 the bird's-eye view of the circuit for QPE, the (quantum) phase estimation algorithm, a standard primitive in many quantum algorithms. QPE is *parametrized* by $n$ (a number of wires) and $U$ (a unitary *oracle*) and is built as follows. First, a register of $n$ qubits is initialized in state $|0\rangle$, while another one is initialized in state $|v\rangle_n$. Then comes the circuit itself: a structured sequence of quantum gates, using the unary Hadamard gate $H$, the circuits $U^{2^i}$ (realizing $U$ to the power $2^i$) and the reversed Quantum Fourier Transform $\texttt{inverse}(\text{QFT }(n))$ . Sub-circuits $U^{2^i}$ and $\texttt{inverse}(\text{QFT }(n))$ are both defined in a similar way.

Here, one should simply note two things: (1) the circuit is made of parallel compositions of Hadamard gates and of sequential compositions of controlled $U^{2^i}$ (the controlled operation is depicted with vertical lines and symbol $\bullet$); (2) the circuit is *parametrized* by $n$ and by $U$. This is very common: in general, a quantum algorithm constructs a circuit whose size and shape depend on the parameters of the problem. It describes a *family of quantum circuits.*

**2.3 Reasoning on circuits and the matrix semantics.** Quantum circuits essentially describe unitary operators [50] acting on Hilbert spaces. In finite dimension, unitary matrices faithfully represent unitary operators: it has been the original mathematical formalism for circuits – coined here as the *matrix semantics.* If this representation is well-adapted for representing simple high-level circuit combinators such as the action of control or inversion, it is not well-suited for specifying the behavior of many complex circuits coming from the literature. Because of this cumbersomeness, textbook descriptions of circuits make use of an informal representation: operators are described by their action on a basis vector (see, for example the description of Shor-OF in [50, p. 232]). This is however understood as a shortcut notation for matrices which remains the main medium for reasoning on circuits. Formal approaches to quantum computation [35,34,53,58,68,71,46,69,45] witness this prevalence of matrices as circuit representation.

**2.4 Path-sum representation.** Path sums [1,2] are a recent symbolic representation. Its strength is to formalize the notation used in quantum algorithm literature (eg, [50]). A unitary operator $U$ is written as $U : |x\rangle \mapsto PS(x)$ where $x$ is a bit vector and $PS(x)$ is defined with the syntax of Fig. 3. In the Figure, addition and multiplication over real are denoted rescpectively with $+$ and $\cdot$, and $x_{[i]}$ is the $i^{th}$ projection of bit vector $x$. The term $n$ is an integer index, characterizing the *range* of the path-sum. Then each term $k \in [\![0, 2^n[\![$ in the path-sum is defined through:

1. the *phase polynomial* $P_k(x)$ – a real value building complex scalar $e^{2\cdot\pi\cdot i\cdot P_k(x)}$;
2. the *basis-ket* function $\phi_k(x)$, defining the ket-vector $|\phi_k(x)\rangle$ this scalar value applies to.

This representation is closed under functional composition and Kronecker product. For instance, if $U$ is defined as in Fig. 3 and if $V$ sends $y$ to $PS'(y)$ defined

$$
\begin{aligned}
PS(x) \;&::= \frac{1}{\sqrt{2}^{n}} \sum_{k=0}^{2^{n}-1} e^{2\cdot\pi\cdot i\cdot P_k(x)} |\phi_k(x)\rangle \\
P(x) \;&::= \frac{x_{[j]}}{2^k} \mid P(x)\cdot P(x) \mid P(x)+P(x) \\
|\phi(x)\rangle \;&::= |b_{[1]}(x)\rangle \otimes \ldots \otimes |b_{[n]}(x)\rangle \\
b_{[j]}(x) \;&::= x_{[j']} \mid \neg b_{[j']}(x) \mid b_{[j']}(x) \;\wedge b_{[j'']}(x) \mid b_{[j']}(x) \;\texttt{XOR}\; b_{[j'']}(x) \mid \texttt{tt} \mid \texttt{ff}
\end{aligned}
$$

Fig. 3: Syntax for regular path-sums [2,1]

as $\frac{1}{\sqrt{2}^{n'}} \sum_{k=0}^{2^{n'}-1} e^{2\cdot\pi\cdot i\cdot P'_k(y)} |\phi'_k(y)\rangle$, then $U \otimes V$ sends $|x\rangle \otimes |y\rangle$ to

$$
\frac{1}{\sqrt{2}^{n+n'}} \sum_{j=0}^{2^{n+n'}-1} e^{2\cdot\pi\cdot i(P_{j/2^n}(x)+P'_{j\%2^n}(y))} |\phi_{j/2^n}(x)\rangle \otimes |\phi'_{j\%2^n}(y)\rangle \tag{1}
$$

which is in the form shown in Figure 3. The compositionality of this semantics is used by Amy [2] to prove the equivalence of large circuit instances. Nonetheless, its main limitation stands in the fact that path-sums only address fixed-size circuits. Albeit a compositional tool, useful to automate proofs, it cannot be used for proving properties of parametrized circuit-building quantum programs.

*This paper proposes an extension of path-sum semantics to address the parametric verification of quantum programs.*

### 3 Introducing PPS

In this section, we introduce the main logical apparatus of our framework: parametrized path-sums. We first present a motivating example and then discuss the construction.

**3.1 Motivating example.** Let us consider the $n$-indexed family of circuits consisting of $n$ Hadamard gates, in sequence, as shown in Figure 4. Sequencing two Hadamard gates can easily be shown equivalent to the identity operation. In other word, when fed with $|0\rangle$, if $n$ is even the circuit outputs $|0\rangle$. Albeit small, this circuit family together with its simple specification exemplifies the typical framework we aim at in the context of certification of quantum programs.

A circuit $C_n$ defined as

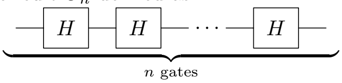

Precondition: $n \geq 0$ is even.

Post-conditions: $\begin{cases} C_n \text{ sends } |x\rangle \text{ to } |x\rangle \\ C_n \text{ consists of } n \text{ gates.} \end{cases}$

Fig. 4: Motivating Example

– The description of the circuit family is parametrized by a classical parameter (here, the non-negative integer $n$);

– The pre-condition imposes both constraints (here, the evenness of $n$) and soundness conditions (here, the non-negativeness of $n$) on the parameters;
– The post-condition can both refer to the semantics of the circuit result and to its form and shape (here, its size).

The circuit family presented in Figure 4 will be used in the rest of the paper as a running, toy example for QBRICKS. In particular, we show in Example 1 how to code it in our framework and how to express the specification in Example 4. Its parametrized implementation in QBRICKS is three lines of code long and its specifications takes six lines. It is proved by recurrence over the parameter $n$, the induction step requiring two calls for lemmas (depending on the evenness of parameter $n$).

**3.2 Parametrizing path-sums.** In order to formalize the semantics of the example of Fig. 4, we aim at generalizing path-sums.

***Illustration.*** For a fixed $n$, the circuit $C_n$ implements either the identity (when $n$ is even), in which case the path-sum is $PS_{Id}(x) = \frac{1}{\sqrt{2}^0} \sum_{k=0}^{2^0-1} e^{2i\pi\cdot 0}|x\rangle$ or the Hadamard gate (when $n$ is odd), in which case the path sum is $PS_H(x) = \frac{1}{\sqrt{2}^1} \sum_{k=0}^{2^1-1} e^{2i\pi\frac{k\cdot x}{2}}|k\rangle$ A candidate parametric path-sum for the family of circuits $\{C_n\}_n$ from Figure 4 could then be written in factorized form as

$$PS_n(x) = \frac{1}{\sqrt{2}^{n\%2}} \sum_{k=0}^{2^{n\%2}-1} e^{2i\pi\frac{(n\%2)\cdot k\cdot x}{2}} |\texttt{if}\ \texttt{even}(n)\ \texttt{then}\ x\ \texttt{else}\ k\rangle. \qquad (2)$$

***Generalization.*** In general, parametrized Path Sums (PPS) are defined over a language of typed terms with possibly free (typed) variables. At the very minimum the language has to be equipped with Boolean values (to handle the values of the ket-vector) and integers (for instance to handle the range).

Given such a language, a PPS is a path-sum where the range, the phase polynomial and the basis-ket can in general be explicit, open terms referring to external parameters. Formally, a `pps` is defined as a function inputting a set of parameters $\overline{p}$ and outputting:

– a parametrized integer $\texttt{pps\_width}(h,\overline{p})$, featuring the number of qubits the target circuit is acting on — its *width*;
– another parametrized integer $\texttt{pps\_range}(h,\overline{p})$, abbreviated as $\texttt{r}(h,\overline{p})$. It indicates the *range* of the sum (defined as the set $\mathrm{BV}_{\texttt{r}(h,\overline{p})}$ of bit vectors of length $\texttt{r}(h,\overline{p})$);
– a *basis ket function* $\texttt{pps\_ket}(h,\overline{p})$, generalizing term $\phi$ from Table 3. For any pair $(x,y)$, of a bit vector $x$ of length $\texttt{pps\_width}(h,\overline{p})$ (standing for an input basis vector) and a bit vector of length $\texttt{r}(h,\overline{p})$, it returns a bit vector of length $\texttt{pps\_width}(h,\overline{p})$ (standing for an output basis vector);
– a parametrized *angle function* $\texttt{pps\_angle}(h,\overline{p})(x,y)$, generalizing the phase polynomial $P$ from Table 3. For any pair $(x,y)$ such as above, it returns a real value $\theta$.

Then, the behaviour of a parametrized quantum circuit $C(\overline{p})$ is described as the *i/o* function inputting a basis ket $|x(\overline{p})\rangle$ of length the width of $C(\overline{p})$ and outputting the parametrized sum term:

$$\texttt{pps\_apply}(h,\overline{p})(|x(\overline{p})\rangle) = \frac{1}{\sqrt{2}^{\texttt{r}(h,\overline{p})}} \sum_{y \in \mathrm{BV}_{\texttt{r}(h,\overline{p})}} e^{2i\pi * \texttt{pps\_angle}(h,\overline{p})(x,y)} |\texttt{pps\_ket}(h,\overline{p})(x,y)\rangle$$

For sake of readability, we often ommit the explicit mention of the parameters. For instance, the PPS $P$ induced by (2) is parametrized by the integer $n$. It is such that for any `int` $n$, $\texttt{pps\_width}(P,n) = 1$ and $\texttt{pps\_range}(P,n) = n\%2$. Furthermore for any bit vectors $x, y$ of lengths 1 and $n\%2$, $\texttt{pps\_ket}(P,n)(x,y)$ is equal to $x$ if $n$ is even and to $y$ otherwise, and $\texttt{pps\_angle}(P,n)(x,y) = n\%2 \cdot x_{[0]} \cdot y_{[0]}$. One then gets expression (2) by applying $\texttt{pps\_apply}(P,\overline{p})$.

Hence, the term language needed for describing PPS of otherwise sophisticated families of quantum circuits can afford to be minimal: first-order typed terms equipped with an equational theory are enough. We also find out that first-order, predicate logic is suitable for specifying the properties of quantum programs: there is no need for higher-order logic such as the ones of Coq or Isabelle/HOL. This is the key to automation.

### 4 Qbricks-DSL

Qbricks-DSL is the (domain-specific) language of our framework. It is designed as a first-order, functional language aimed at *circuit description.* Measurement is out of the scope of the language, and all Qbricks-DSL expressions are terminating. We follow a very simple strategy for circuit building: we use a regular inductive datatype for circuits, where the data constructors are elementary gates, sequential and parallel composition, and ancilla creation. In particular, unlike Quipper [30] or Qwire [53], a quantum circuit is not a function acting on qubits: it is a simple, static object. Nonetheless, as illustrated by our experimentations (Section 8), this does impede neither expressiveness nor parametricity.

Furthermore, even if the language does not feature measurement, it is nonetheless possible to *reason* on probabilistic outputs of circuits, if we were to measure the result of a circuit. Indeed, this can be expressed in a regular theory of real and complex numbers (See Section 6.5).

**4.1 Syntax of Qbricks-DSL.** Qbricks-DSL is a small first-order functional, call-by-value language with a special datatype `circ` as the medium to build and manipulate circuits. The core of Qbricks-DSL can be presented as the simply-typed calculus presented in Figure 6. The basic data constructors for `circ` are `CNOT`, `SWAP`, `ID`, the Hadamard superposition gate `H`, phase shift gate $\texttt{Ph}(e)$ and the parametrized rotation $\texttt{R}_z(e)$. The constructors for high-level circuit operations are sequential composition `SEQ`, parallel composition `PAR` and ancilla creation/termination `ANC` (see Figure 5 for details).

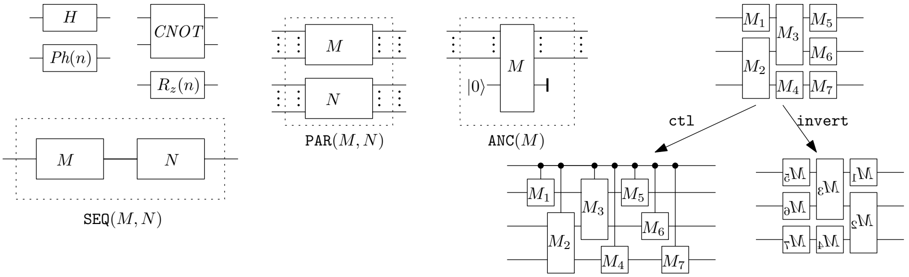

Fig. 5: Circuit combinators

| | | |
|---|---|---|
| Expression | $e$ | $::= x \mid c \mid f(e_1,\dots,e_n) \mid \mathtt{let}\,\langle x_1,\dots,x_n\rangle = e\,\mathtt{in}\,e' \mid$ |
| | | $\mathtt{if}\,e_1\,\mathtt{then}\,e_2\,\mathtt{else}\,e_3 \mid \mathtt{iter}\ f\ e_1\ e_2$ |
| Data Constructor | $c$ | $::= \underline{n} \mid \mathtt{tt} \mid \mathtt{ff} \mid \langle e_1,\dots,e_n\rangle \mid \mathtt{CNOT} \mid \mathtt{SWAP} \mid \mathtt{ID} \mid \mathtt{H} \mid \mathtt{Ph}(e) \mid \mathtt{R}_z(e) \mid$ |
| | | $\mathtt{ANC}(e) \mid \mathtt{SEQ}(e_1,e_2) \mid \mathtt{PAR}(e_1,e_2)$ |
| Function | $f$ | $::= f_{\mathrm{d}} \mid f_{\mathrm{c}}$ |
| Declaration | $d$ | $::= \mathtt{let}\ f_{\mathrm{d}}(x_1,\dots,x_n) = e$ |
| Type | $A$ | $::= \mathtt{bool} \mid \mathtt{int} \mid \top \mid A_1 \times \cdots \times A_n \mid \mathtt{circ}.$ |
| Value | $v$ | $::= x \mid \underline{n} \mid \mathtt{tt} \mid \mathtt{ff} \mid \langle v_1,\dots,v_n\rangle \mid$ |
| | | $\mathtt{CNOT} \mid \mathtt{SWAP} \mid \mathtt{ID} \mid \mathtt{H} \mid \mathtt{Ph}(\underline{n}) \mid \mathtt{R}_z(\underline{n}) \mid \mathtt{ANC}(v) \mid \mathtt{SEQ}(v_1,v_2) \mid \mathtt{PAR}(v_1,v_2)$ |
| Context | $C[-]$ | $::= [-] \mid f(v_1,\dots v_{i-1}, C[-], e_{i+1},\dots,e_n) \mid$ |
| | | $\mathtt{let}\,\langle x_1,\dots,x_n\rangle = C[-]\,\mathtt{in}\,e' \mid \mathtt{if}\,C[-]\,\mathtt{then}\,e_2\,\mathtt{else}\,e_3 \mid$ |
| | | $\mathtt{iter}\ f\ C[-]\ e \mid \mathtt{iter}\ f\ v\ C[-] \mid \langle v_1,\dots v_{i-1}, C[-], e_{i+1},\dots,e_n\rangle \mid$ |
| | | $\mathtt{CNOT} \mid \mathtt{ID} \mid \mathtt{H} \mid \mathtt{Ph}(C[-]) \mid \mathtt{R}_z(C[-]) \mid \mathtt{ANC}(C[-]) \mid$ |
| | | $\mathtt{SEQ}(C[-],e) \mid \mathtt{SEQ}(v,C[-]) \mid \mathtt{PAR}(C[-],e) \mid \mathtt{PAR}(v,C[-])$ |

Fig. 6: Syntax for QBRICKS-DSL

On top of `circ`, the type system of QBRICKS-DSL features the type of integers `int` (with constructors $\underline{n}$, one for each integer $n$), the type of Booleans `bool` (with constructors `tt` and `ff`) and the type of $n$-ary products (with constructor $\langle e_1,\dots,e_n\rangle$). This type system is not meant to be exhaustive and it can be extended with usual constructs such as floats, lists and other user-defined inductive datatypes — its embedding into WhyML makes it easy to use such types. The term constructs are limited to function calls, `let`-style composition, test with `if-then-else` and simple iteration: `iter` $f\ n\ a$ stands for $f(f(\cdots f(a)\cdots))$, with $n$ calls to $f$. We again stress that this could easily be extended — we just do not need it.

The language is first-order: this is reflected by the types $A$ of expressions. The type of a function is given by the types of its arguments and the type of its output. The type of a function with inputs of types $A_i$ and output of type $B$ is written $A_1 \times \cdots \times A_n \to B$.

A function $f$ is either a function $f_{\mathrm{d}}$ defined with a declaration $d$ or a constant function $f_{\mathrm{c}}$. The functions defined by declarations must not be mutually recursive: this small, restricted language only features iteration. Constant functions consist in integer operators ($+$, $*$, $-$, *etc*), Boolean operators ($\wedge$, $\vee$, $\neg$, $\to$, *etc*),

$$\frac{}{\Gamma, x : A \vdash x : A} \qquad \frac{\Gamma \vdash f : A_1 \times \cdots \times A_n \to B \qquad \Gamma \vdash e_i : A_i}{\Gamma \vdash f(e_1, \ldots, e_n) : B}$$

$$\frac{\Gamma \vdash e_i : A_i}{\Gamma \vdash \langle e_1, \ldots, e_n \rangle : A_1 \times \cdots \times A_n}$$

$$\frac{\Gamma \vdash e_1 : A_1 \times \cdots \times A_n \qquad \Gamma, x_1 : A_1, \ldots, x_n : A_n \vdash e_2 : B}{\Gamma \vdash \texttt{let}\, \langle x_1, \ldots, x_n \rangle = e_1 \,\texttt{in}\, e_2 : B}$$

$$\frac{\Gamma \vdash e_1 : \texttt{bool} \quad \Gamma \vdash e_2 : A \quad \Gamma \vdash e_3 : A}{\Gamma \vdash \texttt{if}\, e_1 \,\texttt{then}\, e_2 \,\texttt{else}\, e_3 : A} \qquad \frac{f : A \to A \quad \Gamma \vdash e_1 : \texttt{int} \quad \Gamma \vdash e_2 : A}{\Gamma \vdash \texttt{iter}\ f\ e_1\ e_2 : A}$$

Fig. 7: Typing rules for Qbricks-DSL

comparison operators ($<, \leq, \geq, >, =, \neq : \texttt{int} \times \texttt{int} \to \texttt{bool}$) and high-level circuit operators: $\texttt{ctl}, \texttt{invert} : \texttt{circ} \to \texttt{circ}$ for controlling and inverting circuits, and $\texttt{width}, \texttt{size} : \texttt{circ} \to \texttt{int}$ for counting the number of input and output wires, and the number of gates (not counting `ID` nor `SWAP`) in the circuit $C$. See Figure 5 for the intuitive definition of circuit combinators.

The typing rules are the usual ones, summarized for convenience in Table 7.

**4.2 Operational semantics.** As any other regular functional programming language, Qbricks-DSL is equipped with an operational semantics based on beta-reduction and substitution. We define a notion of value and applicative context as in Fig. 6. We then define a rewriting strategy as the relation defined with $C[e] \to C[e']$ whenever $e \to e'$ is one of the rule of Table 8. The table is split into the rules for the language constructs and the rules defining the behavior of the constant functions. We only give a subset of the latter rules. For instance, the arithmetic operations are defined in a canonical manner, and the Boolean and comparison operators are defined in a similar manner on values of type `int` and `bool`. The rules for the constant functions acting on circuits are also for the most part straightforward: the size of a sequence is the sum of the sizes of the compounds for instance. The rules which we do not provide are the ones for the control operation `ctl`: the intuition behind their definition can be found in [13]. For the elementary gates, any definition can be used (see e.g. [50]), as long as it can be written with the chosen set of gates. One just has to adjust the lemmas referring to `ctl` in Qbricks-Spec. Similarly, the inverse of elementary gates are not given: we can choose the usual ones from the literature —and this definition is then parametrized by the choice of gates.

**4.3 Properties.** The targeted low-level representation for an expression of type `circ` is a value made of the circuit data constructors presented in Table 6: a value $v$ of type `circ` is made out of the grammar $\texttt{SEQ}(v_1, v_2) \mid \texttt{ANC}(v) \mid \texttt{PAR}(v_1, v_2) \mid \texttt{CNOT} \mid \texttt{SWAP} \mid \texttt{ID} \mid \texttt{H} \mid \texttt{Ph}(\underline{n}) \mid \texttt{R}_z(\underline{n})$. Since recursions are reduced to finite iterations, we can derive the following lemma through a simple inductive reasoning:

| Language constructs | |
|---|---|
| Assuming that there is a declaration $f(x_1, \ldots, x_n) \triangleq e$. | |
| $f(v_1, \ldots, v_n) \to e[x_1 := v_1, \ldots, x_n := v_n]$ | |
| $\texttt{let}\ \langle x_1, \ldots, x_n \rangle = \langle v_1, \ldots, v_n \rangle\ \texttt{in}\ e \to e[x_1 := v_1, \ldots, x_n := v_n]$ | |
| $\texttt{if tt then}\ e_1\ \texttt{else}\ e_2 \to e_1$ | |
| $\texttt{if ff then}\ e_1\ \texttt{else}\ e_2 \to e_2$ | |
| when $n \leq 0$: $\texttt{iter}\ f\ \underline{n}\ a \to a$ | |
| when $n > 0$: $\texttt{iter}\ f\ \underline{n}\ a \to f(\texttt{iter}\ f\ \underline{n-1}\ a)$ | |
| **Constant functions (subset of the rules)** | |
| $\underline{n} + \underline{m} \to \underline{n+m}$ | $\texttt{width(CNOT)} \to 2$ |
| $\underline{n} - \underline{m} \to \underline{n-m}$ | $\texttt{width(SWAP)} \to 2$ |
| $\underline{n} * \underline{m} \to \underline{n*m}$ | $\texttt{width}(g) \to 1 \quad (g \text{ other gate})$ |
| $\texttt{size(ID)} \to 0$ | $\texttt{width(SEQ}(v_1, v_2)) \to \texttt{width}(v_1)$ |
| $\texttt{size(SWAP)} \to 0$ | $\texttt{width(PAR}(v_1, v_2)) \to \texttt{width}(v_1) + \texttt{width}(v_2)$ |
| $\texttt{size}(g) \to 1 \quad (g \text{ other gate})$ | $\texttt{width(ANC}(v)) \to \texttt{width}(v) - 1$ |
| $\texttt{size(SEQ}(v_1, v_2)) \to \texttt{size}(v_1) + \texttt{size}(v_2)$ | $\texttt{invert(SEQ}(v_1, v_2)) \to \texttt{SEQ(invert}(v_2), \texttt{invert}(v_1))$ |
| $\texttt{size(PAR}(v_1, v_2)) \to \texttt{size}(v_1) + \texttt{size}(v_2)$ | $\texttt{invert(PAR}(v_1, v_2)) \to \texttt{PAR(invert}(v_1), \texttt{invert}(v_2))$ |
| $\texttt{size(ANC}(v)) \to \texttt{size}(v)$ | $\texttt{invert(ANC}(v)) \to \texttt{ANC(invert}(v))$ |

Table 8: Operational semantics for QBRICKS-DSL

**Lemma 1 (Safety properties and normalization).** *Provided that $\vdash e : A$ is a closed expression, and provided that all the functions in e recursively admit (external) definitions, then either e is a value or it reduces. If $\Gamma \vdash e : A$ and $e \to e'$, then $\Gamma \vdash e' : A$. Finally, the reduction strategy ($\to$) is normalizing: there does not exist an infinite reduction sequence $e_1 \to e_2 \to \ldots$* □

*Example 1.* The example of Section 3.1 can be written in QBRICKS-DSL as

$$\texttt{let aux}(x) = \texttt{SEQ}(x, \texttt{H})$$
$$\texttt{let main}(n) = \texttt{iter aux}\ n\ \texttt{ID}$$

The function `aux` inputs a circuit and appends a Hadamard gate at the end. The function `main` then inputs an integer parameter `n` and iterates the function `aux` to obtain `n` Hadamard in sequence. In particular, one can show that for instance

$$\texttt{main}\ \underline{4} \to^* \texttt{SEQ(SEQ(SEQ(SEQ(ID,H),H),H),H)},$$

that is, a sequence of 4 Hadamard gates.

**4.4 Universality and usability of the chosen circuit constructs.** A *universal* (resp. *pseudo-universal*) set of elementary gates is such that they can be composed thanks to sequence or parallelism so as to perform (resp. approach arbitrarily close) any unitary matrix. In QBRICKS-DSL, we chose the small pseudo-universal elementary set $\{\texttt{CNOT}, \texttt{SWAP}, \texttt{ID}, \texttt{H}\} \cup \bigcup_{\underline{n} \in \mathbb{N}} \{\texttt{Ph}(\underline{n}), \texttt{R}_z(\underline{n}), \}$. Other gates can then be defined as macros on top of them. If one aims at using QBRICKS inside a verification compilation tool-chain, these macros can for instance be the gates of the targeted architecture.

**4.5 Validity of circuits.** A circuit is represented as a rigid rectangular shape with a fixed number of input and output wires. In particular, there is a notion of validity: a `circ` object only makes sense provided two constraints:

- in $\texttt{SEQ}(C_1, C_2)$, the two circuits $C_1$ and $C_2$ should have the same width. For instance, $\texttt{SEQ}(\texttt{CNOT}, \texttt{H})$ is not valid. This is a simple *syntactic* constraint;
- in $\texttt{ANC}(C)$, the circuit $C$ should have $n+1$ wires. Moreover, if given as input a vector where the last qubit is in state $|0\rangle$, its output should also leave this qubit in state $|0\rangle$. This condition is, on the other hand, a *semantic* constraint.

Note that even these syntactic constraints cannot be checked by a simple typing procedure, because of the higher-order reasoning involved here: the constraints must hold for any value of the parameters. All these constraints apply on parametrized circuits. They translate into constraints for the parameters of their related PPS and are expressed in our domain-specific logical specification language, Qbricks-Spec. They are meant to be sent as proof obligations to a proof engine.

*Example 2.* Note how the circuit generated by `main` in Example 1 is not necessarily a valid circuit (although in this case it is). This is one of the constraints that can be handled by Qbricks-Spec, as shown in Example 4.

**4.6 Denotational semantics.** As all expressions in Qbricks-DSL are terminating, one can use regular sets as denotational semantics for the language. In order to be able to handle the definitions coming up in Section 5, we include in the denotation of each type an "error" element $\perp$ We therefore define the denotation of basic types as the set of their values: $[\![\texttt{bool}]\!] = \{\texttt{tt}, \texttt{ff}, \perp\}$, $[\![\texttt{int}]\!] = \mathbb{Z} \cup \{\perp\}$ and $[\![\texttt{circ}]\!] = \{v \mid \vdash v : \texttt{circ}\} \cup \{\perp\}$. Product types are defined as the set-product: $[\![A_1 \times \cdots \times A_n]\!] = ([\![A_1]\!] \times \cdots \times [\![A_n]\!]) \cup \{\perp\}$ and $[\![\top]\!] = \{\star, \perp\}$, the singleton set. Finally, functions are defined as set-functions from the input set to the output set. The denotation of the language constructs are the usual one in a semantics based on sets ; for the constant functions, the definitions are the canonical ones: arithmetic operations maps to arithmetic operations for instance. In Qbricks-DSL, everything is well-defined and $\perp$ is only attainable from $\perp$. For instance, $\perp + x = \perp$.

Note that in the denotational semantics one can build non-valid circuits. For instance, the circuit $\texttt{SEQ}(\texttt{CNOT}, \texttt{H})$ is a member of $[\![\texttt{circ}]\!]$. This is to be expected as we have the following property:

**Lemma 2 (Soundness).** *Provided that* $\vdash e : A$*, we have* $[\![e]\!] \in [\![A]\!] \setminus \{\perp\}$*. Moreover, provided that* $e \to e'$ *then we have* $[\![e]\!] = [\![e']\!]$*.* □

It is however possible to formalize the notion of syntactically valid circuits as a subset of $[\![\texttt{circ}]\!]$.

**Definition 1.** We define the (syntactic) unary relation $\mathcal{V}_{\text{syntax}}$ on $[\![\texttt{circ}]\!]$ as follows: Each one of the gates belongs to $\mathcal{V}_{\text{syntax}}$; if $C_1$ and $C_2$ belongs to $\mathcal{V}_{\text{syntax}}$ then so does $\texttt{PAR}(C_1, C_2)$; if moreover $2 \leq [\![\texttt{width}]\!](C_1)$ then $\texttt{ANC}(C_1)$ belongs to $\mathcal{V}_{\text{syntax}}$ and if $[\![\texttt{width}]\!](C_1) = [\![\texttt{width}]\!](C_2)$ then $\texttt{SEQ}(C_1, C_2)$ belongs to $\mathcal{V}_{\text{syntax}}$.

## 5 Qbricks-Spec

The language Qbricks-DSL is only aimed at manipulating circuits. The reasoning features of Qbricks —and the PPS introduced in Section 3— are defined in the logic and the specification tools offered within Qbricks-Spec.

**5.1 Syntax of Qbricks-Spec.** We define Qbricks-Spec as a first-order, predicate logic with the following syntax.

$$\begin{array}{lll} \text{Formula} & \phi, \psi ::= & \phi \vee \psi \mid \phi \wedge \psi \mid \neg\phi \mid \phi \rightarrow \psi \mid \\ & & R(\hat{e}_1, \ldots, \hat{e}_n) \mid \hat{e}_1 = \hat{e}_2 \\ \text{First-order expression} & \hat{e} \quad ::= & x \mid c(\hat{e}_1, \ldots, \hat{e}_n) \mid f(\hat{e}_1, \ldots, \hat{e}_n) \mid f_\ell(\hat{e}_1, \ldots, \hat{e}_n). \end{array}$$

The first-order expressions $\hat{e}$ form a subset of Qbricks-DSL: they are restricted to variables and (formal) function calls to other first-order expressions. Unlike regular, general expressions —meant to be computational vehicles— these first-order expressions only aim at being reasoned upon. The function names are then expanded with counterpart *logical functions* $f_\ell$. Among these new functions, we introduce one function $\texttt{iter}_f : \texttt{int} \times A \rightarrow A$ for each function $f : A \rightarrow A$, standing for the equational counterpart of the iteration[5]. The logic functions are defined equationally in the logic: see Section 6.4 for details. The relation $R$ ranges over a list of constant relations over first-order expressions. In Qbricks-Spec, we identify relations and functions of return type `bool`. A special relation is the equality: we explicitly introduce it in the syntax to emphasize the fact that Qbricks-Spec is meant to deal with equational theories.

The type system of Qbricks-Spec is extended with opaque types, equipped with constant functions and relations to reason upon them. They come with no computational content: the aim is purely to be able to *express and prove specification properties* of programs. This is why we do not incorporate them in Qbricks-DSL's type system.

The opaque types we consider in Qbricks-Spec are `complex`, `real`, `pps`, `ket` and `bitvector`. The operators and relations for these new types are given in Table 9. Note that in the rest of the paper we will omit the cast operations `i_to_r` and `r_to_c`. We will also use a declared exponentiation function $[-]^{[-]}$ overloaded with types $\texttt{complex} \times \texttt{int} \rightarrow \texttt{complex}$ and $\texttt{real} \times \texttt{int} \rightarrow \texttt{real}$. For any integer $n$ and boolean $b$, constructor `bv_cst` buildsthe bit vector of length $n$ and constant value $b$. Other functions for types `complex`, `real` and `bitvector` are standard. Types `pps` and `ket` are novel and form the main reasoning vehicle in Qbricks-Spec.

**5.2 The types `pps` and `ket`.** In short, the type `pps` encodes our *parametrized path sum* (PPS) representation for expressions of type `circ` in Qbricks-DSL, while `ket` encodes the notion of ket-vector. As these types are pure reasoning apparatuses, we only need them in Qbricks-Spec and they are defined uniquely through an equational theory.

[5] This is required to stay within the grammar of terms of Qbricks-Spec.

| complex and real | | pps | |
|---|---|---|---|
| $i, \pi$ | : complex | pps_width | : pps → int |
| i_to_r | : int → real | pps_range | : pps → int |
| r_to_c | : real → complex | pps_angle | : pps × bitvector × bitvector → real |
| Re, Im, abs | : complex → real | pps_ket | : pps × bitvector × bitvector → bitvector |
| $e^{\square}$ | : complex → complex | pps_apply | : pps × ket → ket |
| $-_c, +_c, *_c, /_c$ | : complex × complex → complex | pps_equiv | : pps × pps → bool |
| $-_r, +_r, *_r, /_r$ | : real × real → real | circ_to_pps | : circ → pps |
| $\sqrt{-}$ | : real → real | **ket** | |
| **bitvector** | | ket_length | : ket → int |
| bv_length | : bitvector → int | ket_get | : ket × bitvector → complex |
| bv_cst | : int × bool → bitvector | bv_to_ket | : bitvector → ket |
| bv_get | : bitvector × int → bool | $+_k, -_k, \otimes_k$ | : ket × ket → ket |
| bv_set | : bitvector × int × bool → bitvector | $*_k$ | : complex × ket → ket |

Table 9: Primary operators for QBRICKS-SPEC

The type pps is equipped with four opaque accessors: pps_width, pps_width, pps_width, pps_ket and pps_angle acting on pps from Section 3.2 and with the function circ_apply. If path-sums compose nicely, a given linear map does not have a unique representative path-sum (partly due to the fact that phase polynomials are equal modulo $2\pi$). To capture this equivalence, we introduce the constant relation pps_equiv. In order to relate circuits and PPS, we introduce the constant function circ_to_pps: it returns *one possible* PPS that *represents* the input circuit. The chosen PPS is built in a constructive manner on the structure of the circuit. A useful relation is $(-\triangleright-)$ relating a circuit and a PPS: it is defined as $(c \triangleright h) \triangleq \texttt{pps\_equiv}(\texttt{circ\_to\_pps}(c), h)$. Another useful macro is function circ_apply : circ × ket → ket, defined as

$$\texttt{circ\_apply}(C, k) \triangleq \texttt{pps\_apply}(\texttt{circ\_to\_pps}(C), k)$$

The type ket is equipped with standard operations for manipulating ket-vectors (Table 9). bv_to_ket turns a bit vector into a basis ket-vector ; ket_length returns the number of qubits in the ket ; ket_get returns the amplitude of the corresponding basis ket-vector. The other operations are the usual operations on vectors: addition, subtraction, tensors, scalar multiplication.

**5.3 Denotational semantics of the new types.** The denotational semantics of real and complex are respectively the sets $\mathbb{R} \cup \{\perp\}$ and $\mathbb{C} \cup \{\perp\}$, and the denotation of the operators are the canonical ones. As for Section 4.6, $\perp$ maps to $\perp$, so for instance $\perp +_r x = \perp$. The denotation of bitvector is defined as the set of all bit-vectors, together with the "error" element $\perp$. The constant functions are mapped to their natural candidate definition, using $\perp$ as the default result when they should not be defined. So for instance, $[\![\texttt{bv\_cst}]\!](-1, \texttt{tt}) = \perp$. An element of ket is meant to be a ket-vector: we defined $[\![\texttt{ket}]\!]$ as the set of *all* possible ket-vectors $\sum_{i=0}^{2^n} \alpha_n |b_n\rangle_m$, for all possible $m, n \in \mathbb{N}$, $\alpha_n \in \mathbb{C}$ and bit-vectors $b_n$ of size $m$, together with the error element $\perp$. Finally, pps is defined as the set of formal path-sums, as defined in Section 3.2, together with the error element $\perp$. The denotation of the constant functions are defined as discussed in Section 5.2. As an example, $[\![\texttt{pps\_range}]\!]$ returns the range of the corresponding PPS. The map `circ_to_pps` builds a valid PPS out of the input circuit, or $\perp$ if the circuit is not valid.

The defined PPS follows the structure of the circuit. For instance, as shown in Eq. (1) on Page 154 the PPS $\texttt{circ\_to\_pps}(\texttt{SEQ}(C_1, C_2))$ is the sequential composition of the two PPS $\texttt{circ\_to\_pps}(C_1)$ and $\texttt{circ\_to\_pps}(C_2)$. This kind of compositionality is what helps with automation.

**5.4 Regular sequents in Qbricks-Spec.** Formulas in Qbricks-Spec are typed objects —and, as mentioned in Section 5.1 one can identify them with first-order expressions of type `bool`. Due to this correspondence, we shall only say that logic judgments in Qbricks-Spec are well-formed judgments of the form $\Delta \vdash \phi$ where the well-formedness means that $\Delta \vdash \phi : \texttt{bool}$ is a valid typing judgment in Qbricks-DSL. That being said, a well-formed judgment $\Delta \vdash \phi$ is valid whenever it holds in the denotational semantics: for every instantiation $\sigma$ sending $x : A$ in $\Delta$ to $[\![A]\!]$, the denotation $[\![\phi]\!]_\sigma$ is valid. In particular, the (free) variables of $\phi$ can be regarded as universally quantified by the context $\Delta$.

**5.5 Parametricity of PPS.** A regular path-sum is not parametric: it represents *one* fixed functional. So why did we chose $[\![\texttt{pps}]\!]$ to be a set of path-sums? Let us consider an example.

*Example 3.* Consider the motivating example of Section 3.1 and its instantiation in Example 1 on page 159. The function `main` describes a family of circuits indexed by an integer parameter $n$. Now, consider the typing judgment

$$h : \texttt{pps}, n : \texttt{int} \vdash (\texttt{main}(n) \triangleright h) : \texttt{bool}.$$

It can be regarded as a relation between PPS $h$ and integers $n$, valid whenever $h$ represents $\texttt{main}(n)$. Technically, this relation is not quite the graph of a function (since several PPS might match the circuit $\texttt{main}(n)$).

**5.6 Standard matrix semantics and correctness of PPS semantics.** Similarly to the type `pps`, Qbricks is endowed with a (logical) type `matrix` to handle the matrix interpretation of circuits, together with various functions and relations to reason on it. In particular, Qbricks features a function $\texttt{mat\_get} : \texttt{matrix} \times \texttt{int} \times \texttt{int} \to \texttt{complex}$, formalizing the access to a matrix element, and a function $\texttt{circ\_to\_mat} : \texttt{circ} \to \texttt{matrix}$ realizing the matrix corresponding to a circuit. We then formally show, within our framework (proven in Why3), that for any valid circuit $C$ and ket $k$ of length $\texttt{width}(C)$, applying $\texttt{circ\_to\_pps}(C)$ on $k$ is equivalent to multiplying it by $\texttt{circ\_to\_mat}(C)$:

**Theorem 1 (Soundness of PPS wrt matrix semantics).**

$$\begin{aligned} &C : \texttt{circ}, k : \texttt{ket} \vdash \texttt{ket\_length}(k) = \texttt{width}(C) \wedge \texttt{valid}(C) \to \\ &\qquad \texttt{apply\_mat}(\texttt{circ\_to\_mat}\, C, k) = \texttt{pps\_apply}(\texttt{circ\_to\_pps}\, C, k) \end{aligned}$$

### 6 Reasoning on Quantum Programs

Thanks to the logic presented in Section 5.4, it is possible to write QBRICKS-SPEC formulas and to express properties of terms of the restricted syntax of Section 5.1. Provided that the regular sequents are *simple enough*, these can automatically be handled with the use of SMT solvers.

In this section, we define a specific Hoare logic, *Hybrid Hoare Logic (HQHL)*, to express pre- and post-conditions for arbitrary QBRICKS-DSL terms. We then discuss the validity of such judgments and explain how to decompose them into elementary, regular sequents (proof obligations). The claim —backed up by our experiments in Section 8— is that the obtained sequents are in practice *simple enough* to be dealt with automatically.

We do not present all HQHL rules here, but simply aim to give an intuition of how and why one can rely on an automated deductive system to derive QBRICKS-SPEC judgments. The complete set of HQHL rules is presented in [13].

**6.1 HQHL judgments.** In order to be able to express program specifications with pre- and post-conditions, we introduce Hybrid Quantum Hoare Logic (HQHL) sequents of the form $\Delta \Vdash \{\phi\}e\{\psi\} : A$ (we omit the type $A$ when irrelevant or clear). The formula $\psi$ can make use of a reserved free variable `result` of type $A$. Such a sequent is then well-formed provided that $\Delta \vdash \phi : \texttt{bool}$, $\Delta, \texttt{result} : A \vdash \psi : \texttt{bool}$ and $\Delta \vdash e : A$ are valid typing judgments. Note how the reserved free variable `result` is being added to $\Delta$ for typing $\psi$. For convenience, as syntactic sugar we allow indexed variables $\texttt{result}_i$ to stand for the $i$th projection of a tuple.

The validity of an HQHL sequent can be defined semantically, similarly to what was done in Section 5.4: $\Delta \Vdash \{\phi\}e\{\psi\} : A$ is valid whenever it is both well-formed and when for every instantiation $\sigma$ sending $x : A$ in $\Delta$ to $[\![A]\!]$ and sending `result` to $[\![e]\!]$, the denotation $[\![\phi \to \psi]\!]_\sigma$ is valid.

In the following sections, we describe the deduction rules that we rely on in QBRICKS. They are designed to be used in a bottom-up strategy to break down judgments into pieces reasoning on smaller terms. Along the way, there is the need for introducing invariants and assertions. As usual, some of these assertions can be derived by computing the weakest-preconditions: we do not necessarily have to introduce every single one. When attaining a term of the restricted grammar of QBRICKS-SPEC that cannot be further decomposed, one can rely on the rule

$$\frac{\Gamma \vdash \phi \to \psi[\texttt{result} := \hat{e}]}{\Gamma \Vdash \{\phi\}\ \hat{e}\ \{\psi\} : A}\ (\texttt{f-o})$$

to generate a proof obligation as a regular sequent in QBRICKS-SPEC.

**6.2 Deduction rules for term constructs.** Figure 10 presents the deduction rules for the term constructs of QBRICKS-DSL carrying a computational content: iteration, tests, function evaluation, etc. We also present a standard weakening rule (`weaken`) and an example of rule for rewriting: The deduction rule (`eq`) states that whenever two expressions are equal one can substitute one

$$\frac{\Gamma, x \Vdash \{\phi \wedge x \leq 0\}\ e_2\ \{P[x, \texttt{result}]\} \quad \Gamma, x, y \Vdash \{\phi \wedge P[x,y]\}\ f(y)\ \{P[x+1, \texttt{result}]\}}{\Gamma \Vdash \{\phi\}\ \texttt{iter}\ f\ \hat{e}_1\ e_2\ \{P[\hat{e}_1, \texttt{result}]\}}\ (\texttt{iter})$$

$$\frac{\Gamma \Vdash \{P\}e_1\{Q[x_i := \texttt{result}_i\} \quad \Gamma, x_1, \ldots, x_n \Vdash \{Q\}e_2\{R\}}{\Gamma \Vdash \{P\}\texttt{let}\, x_1, \ldots, x_n = e_1\ \texttt{in}\, e_2\{R\}}\ (\texttt{let})$$

$$\frac{\Gamma \Vdash \{P\}e_1\{Q[x := \texttt{result}\} \quad \Gamma, x \Vdash \{Q \wedge x\}e_2\{R\} \quad \Gamma, x \Vdash \{Q \wedge \neg x\}e_3\{R\}}{\Gamma \Vdash \{P\}\texttt{if}\ e_1\ \texttt{then}\, e_2[x := e_1]\ \texttt{else}\, e_3[x := e_1]\{R\}}\ (\texttt{if})$$

$$\frac{\forall i, \Gamma \Vdash \{P\}e_i\{R_i[\texttt{result}]\}}{\Gamma \Vdash \{P\}\langle e_1, \ldots, e_2\rangle\{R_1[\texttt{result}_1] \wedge \cdots \wedge R_n[\texttt{result}_n]\}}\ (\texttt{tuple})$$

$$\frac{f(x_1, \ldots, x_n) \triangleq e \quad \Gamma \Vdash \{P\}e[x_1 := e_1, \ldots, x_n := e_n]\{R\}}{\Gamma \Vdash \{P\}f(e_1, \ldots, e_n)\{R\}}\ (\texttt{decl})$$

$$\frac{\Gamma \vdash P \to P' \quad \Gamma \Vdash \{P'\}\ e\ \{Q'\} : A \quad \Gamma, \texttt{result} : A \vdash Q' \to Q}{\Gamma \Vdash \{P\}e\{Q\} : A}\ (\texttt{weaken})$$

$$\frac{\Gamma \vdash e_1 = e_2 : A \quad \Gamma \Vdash \{P[e_1]\}\ e[e_1]\ \{Q[e_1]\} : A}{\Gamma \Vdash \{P[e_2]\}\ e[e_2]\ \{Q[e_2]\} : A}\ (\texttt{eq})$$

Fig. 10: Deduction rules for Qbricks: HQHL rules for term constructs for the other inside a HQHL judgment. Finally, we can derive from the semantics the usual substitution rules. For instance, provided that $\Gamma, x : A \vdash \psi$ and $\Gamma \vdash \hat{e} : A$ then $\Gamma \vdash \psi[x := \hat{e}]$. Note that in the rules, the first-order expressions of the form $\hat{e}$ are from the restricted grammar of terms of Qbricks-Spec.

**6.3 Deduction rules for pps.** The main tools to relate circuits and PPS are the constant function `circ_to_pps`, its relational counterpart $(- \triangleright -)$, and the declared function `circ_apply`. They can be specified inductively on the structure of the input circuit. The complete set of rules for `circ_to_pps` and $(- \triangleright -)$ can be found in [13].

***Compositionality of* SEQ.** For instance, one can derive the deduction rules for `circ_apply` applied to `SEQ` from Table 11. These rules can be used in a bottom-up manner to derive composable, elementary properties of circuits out of sub-circuits. In the table, we abbreviate `pps_acc(circ_to_pps(−))` with $C_{\texttt{acc}}$, for `acc` $\in$ {`width, range, ket,angle`} and, given two bit vectors $x$ and $y$, $x \cdot y$ denotes their concatenation.

***Example of deduction rule for* HAD.** Using the notations from above, we define the following axiom for function `circ_to_pps` applied to the gate `HAD`:

$$\dfrac{\texttt{Prec-SEQ} \triangleq \begin{array}{l} \Gamma \Vdash \{\phi\}C_1\{\texttt{C\_width}(\texttt{result}, \{p\}) = w\} \\ \Gamma \Vdash \{\phi\}C_1\{\texttt{C\_width}(\texttt{result}, \{p\}) = w\} \end{array}}{\Gamma \Vdash \{\phi\}\texttt{SEQ}(C_1, C_2)\{\texttt{C\_width}(\texttt{result}, \{p\}) = w\}}\ \texttt{SEQ}_w$$

$$\dfrac{\{\texttt{Prec-SEQ}\} \quad \begin{array}{l} \Gamma \Vdash \{\phi_1\}C_1\{\texttt{C\_range}(\texttt{result}, \{p\}) = r_1(\{p\})\} \\ \Gamma \Vdash \{\phi_2\}C_2\{\texttt{C\_range}(\texttt{result}, \{p\}) = r_2(\{p\})\} \end{array}}{\Gamma \Vdash \{\phi_1 \ \wedge \ \phi_2\}\texttt{SEQ}(C_1, C_2)\{\texttt{C\_range}(\texttt{result}, \{p\}) = r_1(\{p\}) + r_2(\{p\})\}}\ \texttt{SEQ}_r$$

$$\dfrac{\{\texttt{Prec-SEQ}\} \quad \begin{array}{l} \Gamma \Vdash \{\phi_1\}C_1\{\texttt{C\_angle}(\texttt{result}, \{p\})(x, y_1) = a_1(\{p\}, x, y_1)\} \\ \Gamma \Vdash \{\phi_1\}C_1\{\texttt{C\_ket}(\texttt{result}, \{p\})(x, y_1) = k_1(\{p\}, x, y_1)\} \\ \Gamma \Vdash \{\phi_2\}C_2\{\texttt{C\_angle}(\texttt{result}, \{p\})(k_1(\{p\}, x, y_1), y_2) \\ \qquad = a_2(\{p\}, x, y_1, y_2)\} \end{array}}{\begin{array}{l} \Gamma \Vdash \{\phi_1 \ \wedge \ \phi_2\}\texttt{SEQ}(C_1, C_2)\{\texttt{C\_angle}(\texttt{result}, \{p\})(x, y_1 \cdot y_2) \\ \qquad = a_1(\{p\}, x, y_1) + a_2(\{p\}, x, y_1, y_2)\} \end{array}}\ \texttt{SEQ}_a$$

$$\dfrac{\{\texttt{Prec-SEQ}\} \quad \begin{array}{l} \Gamma \Vdash \{\phi_1\}C_1\{\texttt{C\_ket}(\texttt{result}, \{p\})(x, y_1) = k_1(\{p\}, x, y_1)\} \\ \Gamma \Vdash \{\phi_2\}C_2\{\texttt{C\_ket}(\texttt{result}, \{p\})(k_1(\{p\}, x, y_1), y_2) \\ \qquad = k_2(\{p\}, x, y_1 \cdot y_2)\} \end{array}}{\Gamma \Vdash \{\phi_1 \ \wedge \ \phi_2\}\texttt{SEQ}(C_1, C_2)\{\texttt{C\_ket}(\texttt{result}, \{p\})(x, y_1 \cdot y_2) = k_2(\{p\}, x, y_1 \cdot y_2)\}}\ \texttt{SEQ}_k$$

Fig. 11: Deduction rules for `circ_apply` on sequence of circuits

$$\dfrac{}{\begin{array}{l} \Gamma, x, y : \texttt{bitvector} \Vdash \\ \left\{ \begin{array}{l} \texttt{bv\_length}(x) = 1 \\ \texttt{bv\_length}(y) = 1 \end{array} \right\} \ \texttt{HAD} \ \left\{ \begin{array}{c} \texttt{C\_width}(\texttt{result}) = 1, \\ \texttt{C\_range}(\texttt{result}) = 1, \\ \texttt{C\_angle}(\texttt{result}, x, y) = x_{[0]} * y_{[0]}, \\ \texttt{C\_ket}(\texttt{result}, x, y) = y \end{array} \right\} \end{array}}$$

*Example 4.* Consider the motivating example of Section 3.1 and its instantiation in Example 1. We can now give a specification to the function `main`, as follows:

$$n : \texttt{int}, m : \texttt{int}, x : \texttt{ket} \Vdash \quad \begin{array}{c} \{n \geq 0 \wedge \texttt{ket\_length}(x) = 1 \wedge n = 2 * m\} \\ \texttt{main}(n) \\ \{\texttt{circ\_apply}(\texttt{result}, x) = x\}\,. \end{array}$$

The fact that `circ_apply` is well-defined implies that $C$ is valid.

**6.4 Equational reasoning.** The SMT solvers we aim at using to discharge proof obligations require equational theories describing how to reason on the constant functions that were introduced. Some of these equational theories, such as bit-vectors and algebraic fields, are standard and well-known in verification. Together with a few properties on square-root, exponentiation, real and imaginary parts, the latter is all we need for `real` and `complex`: in quantum computation, the manipulations of real and complex numbers turn out to be quite limited – we do not need anything related to real or complex analysis.

The main difficulty in the design of QBRICKS has been to lay out equational theories and lemmas for `circ`, `pps` and `ket` that can efficiently help in automatically discharging proof obligations. Many of these equations and lemmas are quite straightforward. For instance, we turn the rewriting rules of Table 8 into equations, such as $(x, y : \texttt{circ}) \vdash \texttt{width}(\texttt{PAR}(x, y)) = \texttt{width}(x) + \texttt{width}(y)$, or $a : A, n : \texttt{int} \vdash \texttt{iter}_f(a, n+1) = f(\texttt{iter}_f(a, n))$. These equations maps the (syntactic) computational behavior of expressions into the logic.

Other equations express purely semantic properties. For instance,

$$\Gamma, k : \texttt{ket} \vdash \texttt{circ\_apply}(\texttt{SEQ}(C_1, C_2), k) = \texttt{circ\_apply}(C_1, \texttt{circ\_apply}(C_2, k)) \quad (3)$$

(together with a few hypotheses ensuring correct widths) can be derived from Table 11 and is part of the equational theory.

**6.5 Additional deductive rules.** QBRICKS provides additional reasoning rules, that we do not have space enough to detail here. Upon them are:

*Circuit complexity.* Certifying the complexity of quantum implementations (e.g., polynomial number of gates in the size of the input) is of primary importance as in mid-term, implementations will have to deal with limited hardware capacities, hence the need for tight circuit constructions. We stress that, while raised by several programming [30] or compilation works [48], this aspect of certification is not addressed by existing formal verification approaches [35,45,1].

*Probabilities.* The probability of obtaining a result by a measurement is correlated with the amplitudes of the corresponding ket-basis vectors in the quantum state of the memory. In QBRICKS-SPEC we define $\texttt{proba\_partial\_measure} : \texttt{circ} \times \texttt{ket} \times \texttt{bitvector} \to \texttt{real}$ meaning that when the input circuit is applied to the input ket, if we were to measure the result the probability of obtaining the given vector would be the result of the function.

*Wire identification.* In some situation, to add a gate in a circuit it is easier to give the number (identifier) of the wire on which the gate applies (such as "apply `HAD` on wire $n$") instead of sequencing the circuit with $\texttt{Id}^{\otimes n-1} \otimes \texttt{HAD}$. This is for instance the design chosen in QASM or SQIR [35].

In QBRICKS it is possible to define such a macro with the use of a derived constructor $\texttt{PLACE}(C, k, n)$. For any circuit $C$ and any integers $k, n$, if $0 \leq k \leq n - \texttt{width}(C)$, $\texttt{PLACE}(C, k, n)$ applies $C$ on wires $k$ to $k + \texttt{width}(C) - 1$. It is defined as $\texttt{ID}^{\otimes k} \otimes C \otimes \texttt{ID}^{\otimes n-k-\texttt{C\_width}(C)}$, where for any $0 < i$, $\texttt{ID}^i \triangleq \texttt{iter par-ID}\ (i-1)\ \texttt{ID}$ and $\texttt{par-ID}(C) \triangleq \texttt{PAR}(C, \texttt{ID})$. Similarly, QBRICKS also provides constructor $\texttt{CONT}(C, c, k, n)$ with additional index $c$ in $[\![0, n[\![$ and not in $[\![k, k + \texttt{width}(c)[\![$. Using adequate qubit permutation, through combinations of `PLACE` and `SWAP`, it applies $\texttt{PLACE}(C, k, n)$ with control $c$.

### 7 Implementation

The framework described so far is implemented as a DSL embedded inside the Why3 deductive verification platform [9,25], written in the WhyML programming language. This allows us to benefit from several strengths of Why3, such as efficient code extraction toward Ocaml, generation of proof obligations (to implement the HQHL mechanism) and access to several proof means: SMT solvers, interactive proof commands or export to proof assistants (Coq, Isabelle/HOL) —although we do not use this latter option in our case-studies.

The development itself counts 17,000+ lines of code, including 400+ definitions and 1700+ lemmas, all proved within Why3. Most of the development concerns the (verified) mathematical libraries. They cover the mathematical structures at stake in quantum computing (complex numbers, Kronecker product, bit-vectors, etc.), together with a *formally verified collection* of mathematical results. Only two theorems are assumed (for any real $x$: if $0 \leq x \leq 1$ then $\texttt{sin}(\pi x) \leq \pi x$, and $x \leq \texttt{sin}(\pi \frac{x}{2})$). Proving them requires function derivation material, not available in Why3 so far. Hence we chose to assume these standard results.

### 8 Case studies and experimental evaluation

We develop and prove parametric implementations of Grover's search, the Quantum Fourier Transform (QFT), the Quantum Phase Estimation (QPE) and the first ever verified implementation of the quantum part of Shor's algorithm (Shor-OF). We also implemented Deutsch-Jozsa (DJ) for comparison.

**8.1 Examples of formal specifications.** Let us first introduce some of the formal specifications we proved. The specification for QPE [41,16] is shown in Figure 12(a). The procedure inputs a unitary operator $U$ and an eigenvector $|v\rangle$ of $U$ and finds the ghost ([26]) eigenvalue $e^{2\pi i \Phi_v}$ associated with $|v\rangle$. The specification for Shor-OF [61] is shown in Figure 12(b). We developed a certified *concrete* implementation following the implementation proposed in [5] —a reference in term of complexity.[6] The specification for Grover [31] is shown in Figure 12(c). Given a predicate with $k$ true value in $[\![0, 2^n[\![$, Grover's algorithm outputs one of these true values with good probability.

Each of these specifications makes use of specific functions that we do not have the space to detail here (see [13] for details). We however want to note two things. First, these specifications describe results of measurement (with the dedicated functions `proba_partial_measure_x`). As discussed in Section 6.5, if Qbricks-DSL is not able to handle measurement we are still able with Qbricks-Spec to reason on the *result* of a measurement, as this is a simple function over complex amplitudes. Another thing to note is that, for Shor-OF and Grover, our specification discuss the *polynomial size* of the produced circuit.

[6] A further refinement is possible [5], using a hybrid version of the Quantum Fourier Transform, but it would require adding effective measure operation and classical control to Qbricks.

$$\Gamma, (f : \texttt{pps}), (C : \texttt{circ}), (|v\rangle : \texttt{ket}), (k, n : \texttt{int}), (\texttt{ghost}\ \theta : \texttt{real}), (j : \texttt{ghost int}) \Vdash$$
$$\big((C \rhd f)\ \wedge\ \texttt{width}(C) = n\ \wedge\ 0 < k\ \wedge\ \texttt{Eigen}(f, |v\rangle, e^{2\pi i * \theta})\big)$$
$$\texttt{QPE}(C, k, n)$$
$$\begin{pmatrix} \texttt{proba\_partial\_measure\_p}(\texttt{result}, k|v\rangle, \texttt{error} < \frac{1}{2^{k+1}}) \geq \frac{4}{\pi^2}\ \wedge \\ \theta = \frac{j}{2^k} \to \texttt{proba\_partial\_measure}(\texttt{result}, |v\rangle, |j\rangle_k) = 1 \end{pmatrix}$$

(a) Specification for our implementation of Quantum Phase estimation

$$\Gamma(a, b, n : \texttt{int}), (j : \texttt{ghost int}) \Vdash$$
$$\big(\texttt{co\_prime}(a, b) \wedge\ 1 \leq b < 2^n \wedge 1 \leq j < b\ \wedge a^j \% b = 1\big)$$
$$\texttt{Shor-circ}(a, r, n)$$
$$\begin{pmatrix} \texttt{proba\_partial\_measure\_p}\big(|1\rangle_n, \texttt{error}_1 \leq \frac{1}{2*2^{n*2}}\ \big) \geq \frac{4}{\pi^2}\ \wedge \\ \texttt{proba\_partial\_measure\_p}\big(|1\rangle_n, \texttt{error}_2 \leq \frac{1}{2*2^{n*2}}\ \big) \geq \frac{\phi(r)}{r} \times \frac{4}{\pi^2} \wedge \\ \texttt{size}(\texttt{result}) = \texttt{Shor-poly}(n)\ \wedge \\ \texttt{ancillas}(\texttt{result}) = n + 2 \wedge\ \texttt{width}(\texttt{result}) = 3 * n \end{pmatrix}$$

(b) Specification for our implementation of Shor-OF algorithm

$$\Gamma, (C : \texttt{circ}), (f : \texttt{int} \to \texttt{bool}), (n, i, k : \texttt{int}) \Vdash$$
$$\begin{pmatrix} \texttt{implements}(C, f)\ \wedge 1 < n\ \wedge 1 \leq k < 2^n - 1\ \wedge 1 \leq i \\ \wedge\ \texttt{Card}(\{j \mid 0 \leq j < 2^n \wedge f(j) = \texttt{true}\}) = k \end{pmatrix}$$
$$\texttt{Grover}(C, k, n)$$
$$\begin{pmatrix} \texttt{proba\_partial\_measure}_f(\texttt{result}, \texttt{bv\_cst}\ \ (n, 0), f) = \sin^2\left(\arcsin\left(\sqrt{\frac{k}{2^n}}\right)\dot{(}1 + 2i)\right)\ \wedge \\ \texttt{size}(\texttt{result}) = i * (\texttt{size}(C) * \mathcal{O}(n))\ \wedge \\ \texttt{width}(\texttt{result}) = n \wedge \texttt{ancillas}(\texttt{result}) = 1 \end{pmatrix}$$

(c) Specification for our implementation of Grover's algorithm

Fig. 12: Specifications of the main implementations

**8.2 Experimental evaluation.** Different metrics about our formal developments are reported in Table 13[7]: lines of decorated code, number of lemmas, proof obligations (PO), automatically proven PO (within time limit 5 seconds) and their percentage among POs, interactive commands we entered to discharge them and time required for the automatic verification of these proofs.

Note that metrics for each implementation strictly concern the code that is *proper* to it (eg., QPE contains calls to QFT but QPE line in Table 13 does not include the QFT implementation. The whole Shor-OF development is reported in the "Shor-OF full".

***Result.*** *Qbricks did allow us to implement and verify in a parametric manner the Shor-OF, QPE and Grover algorithms, at a rather smooth cost and with high proof automation (95% on average, 95% for full Shor-OF).*

**8.3 Prior verification efforts.** Before comparing our approach to prior attempts (Table 14), we first introduce these cases.

[7] Experiments were run on Linux, on a PC equipped with an Intel(R) Core(TM) i7-7820HQ 2.90GHz and 15 GB RAM. We used Why3 version 1.2.0 with solvers Alt-Ergo-2.2.0, CVC 3-2.4.1, CVC4-1.0, Z3-4.4.1.

| | #LoC + Spec | #Extr. | #Def. | #Lem. | #POs | Automation # Aut. | % Aut. | #Cmd | Verif. time |
|---|---|---|---|---|---|---|---|---|---|
| DJ | 57 | 11 | 2 | 1 | 72 | 61 | >84% | 39 | 1m19s |
| Grover | 193 | 39 | 6 | 8 | 505 | 479 | >94% | 125 | 4m43s |
| QFT | 65 | 18 | 3 | 0 | 62 | 53 | >85% | 37 | 1m11s |
| QPE | 175 | 24 | 3 | 8 | 282 | 262 | >92% | 94 | 4m35s |
| Shor-OF | 923 | 132 | 28 | 14 | 2473 | 2386 | >96% | 421 | <18m |
| Shor-OF (full) | 1163 | 174 | 34 | 22 | 2817 | 2701 | >95% | 552 | <23m |
| Total | 1423 | 224 | 42 | 31 | 3394 | 3241 | >95% | 716 | <29m |

##LoC + Spec.: lines of decorated code — # Extr.: lines of extracted code (OCaml)
#Aut.: automatically proven POs — #Cmd: interactive commands
#Verif. time: automated proof verification time

Table 13: Implementation & verification for case studies with Qbricks

***Regular path-sums.*** [2,1] uses path sums for the verification of several circuits of complexity similar to that of QFT (QFT, Hidden shift, generalized Toffoli, etc). Yet, these experiments consider fixed circuits (up to 100 qubits) and the technique cannot be applied to parametric families of circuits or circuit-building languages.

***QHL.*** Liu et al. [45] report about the parametric verification of Grover search algorithm, on a *restricted case* [8] and in the *high-level* algorithm description formalism of QHL – especially QHL has no notion of circuit. So for instance one cannot reason upon the size of a circuit within QHL.

***SQIR.*** Finally, Hietala et al. [35] have presented a parametric (circuit-building) implementation of the Deutsch-Jozsa algorithm in Coq, with two independent full correctness proofs. Recently (Oct. 2020), the authors also presented parametrized versions of QFT, QPE and Grover algorithms [34].

**8.4 Evaluation: benefits of PPS and Qbricks.** So as to evaluate the proof effort gain of using pps instead of matrices, Table 14 shows some comparison between our case studies implementations and equivalent proved implementations from the literature: the Grover algorithm implementation from [45] in Isabelle/HOL and the implementations [35,34] using SQIR and Coq. As supplementary comparison terms, we implemented Qbricks versions of both QFT and Deutsch-Jozsa using exclusively matrices.

For example the Qbricks implementation of QFT with pps is 18 lines long, with 47 lines of specifications and intermediary lemmas, and its proof required 37 additional interactive commands, hence Spec + Cmd = 84. In comparison, the corresponding SQIR development uses 287 interactive commands (7.7x more).

***Conclusion.*** Relying on PPS semantics and first-order logic instead of matrices and higher-order logics strongly eases the proof effort. In term of command

[8] The case in [45, p. 232] concerns cases where the number $k$ of seeked values is equal to $2^j$ for a given integer $j$.

| | Qbricks pps | | | | Qbricks Matrix | | | |
|---|---|---|---|---|---|---|---|---|
| | LoC | Spec | Cmd | Spec+Cmd | LoC | Spec | Cmd | Spec+Cmd |
| DJ | 11 | 46 | 39 | 85 | 11 | 129 | 131(>3.3x) | 260(>3x) |
| QFT | 18 | 47 | 37 | 84 | 18 | 172 | 106(>2.8x) | 278 (>3.3x) |
| Grover | 39 | 154 | 125 | 279 | | | | |
| QPE | 23 | 152 | 94 | 246 | | | | |

| | SQIR | | | | QHL | | | |
|---|---|---|---|---|---|---|---|---|
| | LoC | Spec | Cmd | Spec+Cmd | LoC | Spec | Cmd | Spec+Cmd |
| DJ | 10 | 39 | 222(>5.6x) | 261(>3x) | | | | |
| QFT | 10 | 44 | 287(>7.7x) | 331(>3.9x) | | | | |
| Grover | 15 | 121 | 805(>6.4x) | 926(>3.3x) | 90 | 1263 | 1712(>13.6x) | 2975 (>10.6x) |
| QPE | 40 | 86 | 726(>7.7x) | 812(>3.3x) | | | | |

##LoC.: lines of code – # Spec.: lines of spec. and lemmas – #Cmd: proof commands

Table 14: Compared implementations of case studies, using matrices and pps lines, proofs are consistently at least 5.6x shorter than non Qbricks examples, up to 13.6x for the case of Grover in QHL and 7.7x for QPE and QFT in SQIR.[9]

### 9 Related works

***Formal verification of quantum circuits.*** Prior efforts regarding quantum circuit verification [27,45,70,53,56,1,2,35,34] have been described throughout the paper, especially in Sections 1, 3.1 and 8. Our technique is more automated than those based on interactive proving [35,34,45], borrows and extends the path sum representation [2] to the parametric case, and do consider a circuit-building language rather than a high-level algorithm description language [45].

***Quantum Languages and Deductive Verification.*** Liu *et al.* [45] introduce Quantum Hoare Logic for high-level description of quantum algorithms. QHL and our own HQHL are different, as the underlying formalisms have different focus. While QHL deals with measurement and classical control, it does not allow reasoning on the structure of the circuit. On the other hand, Qbricks does not handle classical control, but it brings better proof automation and deduction rules for reasoning on circuits. Combining the two approaches is an exciting research direction.

***Verified Circuit Optimizations.*** Formal methods and other program analysis techniques are also used in quantum compilation for verifying circuit *optimization* techniques [52,6,32,3,62,57,35]. Epecially, the ZX-calculus [17] represents

[9] The difference with SQIR in the column "Spec+Cmd" is less stringent. By the way, it turns out that SQIR syntax for specifications is often more succint, as eg, Qbricks writes each precondition in a separated line, where Coq writes the same as a single-line conjunction.

quantum circuits by diagrams amenable to automatic simplification through dedicated rewriting rules. This framework leads to a graphical proof assistant [40] geared at certifying the semantic equivalence between circuit diagrams, with application to circuit equivalence checking and certified circuit compilation and optimization [21,20,39]. Yet, formal tools based on ZX-calculus are restricted to fixed circuits, and parametrized approaches are so far limited to pen-and-paper proofs [12].

***Other quantum applications of formal methods.*** Huang *et al.* [36,37] proposes a "runtime-monitoring like" verification method for quantum circuits, with an annotation language restricted to structural properties of interest (e.g., superposition or entanglement). Similarly, [44] describes a projection based assertion language for quantum programs. Verification of these assertions is led by statistical testing instead of formal proofs. The recent Silq language [8] also represents an advance in the way toward automation in quantum programming. It automatizes uncomputation operations, enabling the programmer to abstract from low level implementation details. Also specialized type systems for quantum programming languages, based on linear logic [60,59,43] and dependent types [51,53], have been developed to tackle the non-duplicability of qubits and structural circuit constraints. Finally, formal methods are also at stake for the verification of quantum cryptography protocols [49,29,11,47,19].

### 10 Conclusion

We address the problem of automating correctness proofs of quantum programs. While relying on the general framework of deductive verification, we finely tune our domain-specific circuit-building language Qbricks-DSL together with its new logical specification language Qbricks-Spec in order to keep correctness reasoning over relevant quantum programs within first-order theory. Also, we introduce and intensively build upon *parametrized path sums* (PPS), a symbolic representation for quantum circuits represented as functions transforming quantum data registers. We develop verified parametric implementations of the Shor-OF algorithm (first verified implementation) and other famous non-trivial quantum algorithms (including QPE and Grover search), showing significant improvement over prior attempts – when available.

***Acknowledgments.*** This work was supported in part by the French National Research Agency (ANR) under the research project SoftQPRO ANR17-CE25-0009-02, and by the DGE of the French Ministry of Industry under the research project PIA-GDN/QuantEx P163746- 484124.
